# Draft Genome Sequence of the Enteropathogenic Bacterium *Campylobacter jejuni* Strain cj255

**DOI:** 10.1128/genomeA.01223-15

**Published:** 2015-10-22

**Authors:** Fariha Masood Siddiqui, Muhammad Ibrahim, Nighat Noureen, Zobia Noreen, Richard W. Titball, Olivia L. Champion, Brendan W. Wren, David Studholme, Habib Bokhari

**Affiliations:** a Department of Biosciences, COMSATS Institute of Information Technology, Islamabad, Pakistan; b Department of Biosciences, COMSATS Institute of Information Technology, Sahiwal, Pakistan; c Bacterial Pathogenesis Group, Department Biosciences, College of Life and Environmental Sciences, University of Exeter, Exeter, United Kingdom; d Pathology Molecular Biology Unit, The London School of Hygiene and Tropical Medicine, London, United Kingdom

## Abstract

The enteropathogen *Campylobacter jejuni* is a global health disaster, being one of the leading causes of bacterial gastroenteritis. Here, we present the draft genome sequence of *C. jejuni* strain cj255, isolated from a chicken source in Islamabad, Pakistan. The draft genome sequence will aid in epidemiological studies and quarantine of this broad-host-range pathogen.

## GENOME ANNOUNCEMENT

*Campylobacter jejuni* is a Gram-negative, motile, and microaerophilic pathogen belonging to the *Epsilonproteobacteria*. *C. jejuni* infections are termed as campylobacteriosis or *C. jejuni* gastroenteritis. Campylobacteriosis results in diarrhea, which is frequently watery or bloody (1, 2). Campylobacteriosis is prominently a food-borne illness, which involves foods of livestock or poultry origin, with poultry especially contributing to the disease burden worldwide. This bacterium can survive and multiply in a variety of ecological niches, even under harsh environmental conditions (3, 4). *C. jejuni* shows a broad host range, including poultry, birds, livestock animals, domestic pets, wild animals, and marine vertebrates, which makes them successful pathogens (5).

We sequenced and annotated the *C. jejuni* strain cj255, isolated from a broiler poultry source in the Islamabad, Pakistan suburbs (with extensive poultry farming activity). The genomic DNA, isolated with a Wizard genomic DNA purification kit (Promega, Madison, WI), was whole-genome sequenced using the Illumina HiSeq 2000 sequencing system with sequence depth of 2,395.0× following genome assembly using Velvet version 102.03. The draft assemblies were based on 419,928 total reads resulting in 25 contigs. All libraries provided 30-fold coverage of the genome. Functional annotation was done by analyzing the results obtained from the Rapid Annotations using Subsystems Technology (RAST) server (6), tRNAscan-SE 1.21 (7), and RNAmmer 1.2 (8).

The draft genome sequence of *C. jejuni* strain cj255 comprises 1,634,595 bases, representing approximately >99.9% of the estimated genome, with 25 contigs. The genome of this strain has a G+C content of 31%. A total of 1,711 coding sequences (CDSs) and 39 RNAs were predicted. Among the coding sequences, not only those for conserved regions, such as genes for metabolic, biosynthetic, cellular, and regulatory processes, but also those for several putative virulence determinants, including genes encoding the cytolethal distending toxin, flagellar structural proteins, phospholipase A, the PEB antigenic surface proteins, and proteins potentially involved in host-pathogen interactions, such as Campylobacter invasion antigen (CiaB), *C. jejuni* fibronectin binding protein (CadF), and Chemotaxis protein (CheY) (9, 10), were identified. The most interesting features of cj255 are the presence of type VI secretion systems, like in human strains *C. jejuni* cj1, 00-1597, 00-1597.

Our study has revealed extensive genetic diversity among *C. jejuni* strains and paves the way toward the identification of correlates of pathogenicity and the development of improved epidemiological tools for this problematic pathogen.

### Nucleotide sequence accession numbers.

This whole-genome shotgun project has been deposited at DDBJ/EMBL/GenBank under accession no. ARWS00000000. The version described in this paper is the first version, ARWS00000000.1.
